# A Lower-Class Advantage in Face Memory

**DOI:** 10.1177/01461672221125599

**Published:** 2022-11-05

**Authors:** Pia Dietze, Sally Olderbak, Andrea Hildebrandt, Laura Kaltwasser, Eric D. Knowles

**Affiliations:** 1 New York University, New York City, USA; 2 University of California, Irvine, USA; 3 Universität Ulm, Germany; 4 Carl von Ossietzky Universität Oldenburg, Germany; 5 Humboldt-Universität zu Berlin, Germany

**Keywords:** social class, face memory, relevance appraisals, eyewitness identification

## Abstract

People remember what they deem important. In line with research suggesting that lower-class (vs. higher class) individuals spontaneously appraise other people as more relevant, we show that social class is associated with the habitual use of face memory. We find that lower-class (vs. higher class) participants exhibit better incidental memory for faces (i.e., spontaneous memory for faces they had not been instructed to memorize; Studies 1 and 2). No social-class differences emerge for faces participants are instructed to learn (Study 2), suggesting that this pattern reflects class-based relevance appraisals rather than memory ability. Study 3 extends our findings to eyewitness identification. Lower-class (vs. higher-class) participants’ eyewitness accuracy is less impacted by the explicit relevance of a target (clearly relevant thief vs. incidental bystander). Integrative data analysis shows a robust negative association between social class and spontaneous face memory. Preregistration (Studies 1 and 3) and cross-cultural replication (Study 2) further strengthen the results.

Research suggests that an individual’s social class has pervasive effects on social–cognitive functioning, often at a spontaneous level. Relative to their higher class counterparts, lower-class individuals exhibit heightened neural responses related to empathy (Varnum et al., 2015), increased physiological arousal signaling compassion in response to others’ suffering (Stellar et al., 2012), and more spontaneous attention to other human beings (Dietze & Knowles, 2016). The current study extends this work by examining the relationship between social class and an important facet of social cognition: memory for faces. Face memory, or the ability to learn and recognize persons based on their facial features, is crucial for normal social interaction. However, face memory performance varies across individuals (e.g., Wilhelm et al., 2010) and is sensitive to a range of factors—including developmental disorders (e.g., autism spectrum disorder; Weigelt et al., 2012), the sex of the perceiver (Herlitz et al., 1997; Lewin & Herlitz, 2002), and characteristics of the target person (e.g., in-group/out-group status; Meissner & Brigham, 2001). We theorize that an individual’s social class is an additional factor that can affect face memory performance. Specifically, we hypothesize that lower-class individuals might use face memory ability more habitually than higher-class individuals, leading to a lower-class advantage in *spontaneous* memory for faces.

## Proposed Mechanism: Motivational Relevance

Dietze and Knowles (2016, 2021) theorized that many of the observed social-class differences in social attunement stem from a person’s appraisal of other human beings’ motivational relevance—the degree to which a person sees other people as potentially rewarding, threatening, or otherwise worth paying attention to (Lang et al., 2013). In experimental research, motivationally relevant stimuli are those that activate approach or avoidance responses in the brain because they impinge on the individual’s goals and well-being (e.g., Sander et al., 2003). The result is heightened physiological arousal and more deeply engaged attention (e.g., Bradley et al., 1992; Briggs & Martin, 2009; Schupp et al., 2000).

Importantly, motivational relevance appraisals of a stimulus can be elicited by the immediate context or by factors that an individual brings to the context (i.e., an individual’s priors). People’s priors—the encoding of one’s past experience with a stimulus—may render a stimulus *spontaneously* relevant. In such cases, perceivers are likely to appraise the stimulus as motivationally relevant regardless of the context in which it is presented. For example, individuals show a tendency to better recall same-race faces than other-race faces; this is called the cross-race effect (CRE; also called the own-race bias or the other-race effect; for a review see Meissner & Brigham, 2001). Here, priors the individual brings to the context spontaneously confer relevance on same-race stimuli, leading to better memory for same-race faces (e.g., Hugenberg et al., 2010). In other cases, motivational-relevance appraisals are contingent on the context such that perceivers will only deem a stimulus relevant if features of the context confer relevance on it. In the case of the cross-race effect, instructions that participants should attend closely to the individuating characteristics of the faces (e.g., to avoid racial bias) can eliminate the CRE (Hugenberg et al., 2007). Here, cues in the context (i.e., task instructions) “turn up” the motivational relevance of the stimulus, outweighing the priors an individual brings to the situation; the other-race stimulus previously deemed motivationally irrelevant is now appraised as motivationally relevant and thus, remembered (Bernstein et al., 2007; Hugenberg et al., 2010; Rhodes et al., 2009; Shriver et al., 2008).

We hypothesize that compared with higher-class individuals, lower-class individuals are more likely to enter a new situation with high “social priors”—that is, with the assumption that other human beings are relevant to them. However, we do not predict that higher-class individuals never appraise other humans as motivationally relevant. Instead, we hypothesize that strong cues in the context (e.g., if human beings are rendered goal-relevant by task demands) can elicit relevance appraisals of other human beings regardless of a person’s social class. Thus, we suggest that lower-class individuals, by virtue of their high social priors, appraise others they encounter as relevant *by default*—whereas higher-class individuals require additional contextual cues if they are to appraise others as relevant.

## Social-Class Cultures and Motivational Relevance

Why would lower-class individuals have higher social priors than higher-class individuals? We argue that social priors reflect cultural experiences rooted in the resource ecologies within which members of different social classes develop. Higher-class individuals possess more material resources, reducing their need to rely on others for successful functioning, while lower-class individuals compensate for a lack of material resources by adopting interdependent strategies to meet their needs (Fiske & Markus, 2012; Grossmann & Varnum, 2011; Kraus et al., 2012; Snibbe & Markus, 2005, Stephens et al., 2007). Implicit in the use of such interdependent strategies vis-à-vis social class is the notion that others may frequently promote or hinder one’s goals—that others are typically high in motivational relevance—resulting in the development of high social priors.

Class-based differences in social priors can parsimoniously explain a host of findings regarding social attunement among individuals higher and lower in social class. For instance, research has shown that lower (versus higher) social class is associated with important downstream consequences such as working more efficiently in groups (Dittmann et al., 2020) and more engagement cues during social interaction (Kraus & Keltner, 2009). People with higher social priors would be expected to orient to others more reliably than those who require strong contextual cues to do so.

Not only are motivational relevance appraisals theorized to be the distal mechanism for these outcomes, but they are also theorized to make novel predictions about more spontaneous or basic forms of social information processing. Specifically, the tendency for lower-class individuals to appraise other people as relevant to their current goals and well-being is especially pronounced at the default level—that is, in the absence of, or before one can process, information about a person’s relevance (e.g., when encountering an unknown situation or a stranger). As such, a lower-class (vs. higher-class) person’s heightened relevance appraisals of other human beings are thought to manifest at the earliest stages of information processing—for example, in spontaneous cognition, attention, and memory—through the social priors that an individual brings to that situation (Dietze & Knowles, 2016, 2021).

Research in the domain of visual attention provides evidence for this motivational-relevance hypothesis. Within milliseconds of encountering a stimulus, individuals make an initial appraisal concerning its relevance to our current goals and well-being; more motivationally relevant stimuli garner more attention (Brosch & Van Bavel, 2012; Lang et al., 2013; Leite et al., 2012). Across a number of experiments that included filming pedestrians’ visual field on a busy street and eye-tracking participants in the laboratory, higher-class participants allocate less spontaneous attention to other human beings than their lower-class counterparts (Dietze & Knowles, 2016). Further evidence for the motivational relevance account comes from the domain of Theory of Mind in which social class was found to be negatively associated with the spontaneous ability to infer and use information about others’ mental states (Dietze & Knowles, 2021; Kraus et al., 2010). Specifically, lower-class participants exhibit better spontaneous perspective-taking of another person’s visual perspective and thus perform better on a task that involves moving objects while taking another person’s perspective into account (Dietze & Knowles, 2021). In the present work, we aim to extend the logic of relevance appraisals to memory for faces.

## Face Memory and Relevance Appraisals

While attentional processes documented in past studies make a strong case for the motivational relevance account, to our knowledge no research has yet documented class-based differences in social memory. Like attention, memory is considered an important marker of motivational relevance appraisals in the brain because it signals the depth of processing; simply put, individuals remember what they deem important (e.g., Bradley et al., 1992; e.g., Rodin, 1987; Rule et al., 2007). The present research allows us to test our motivational relevance account in the domain of memory while holding the amount of attention to a stimulus (i.e., a face) constant.

Our study of face memory also allows us to clarify a point of empirical ambiguity present in past research. While past studies have documented a negative correlation between social class and various forms of social cognition, it is not entirely clear that these effects reflect class-based differences in motivation relevance appraisals rather than differences in social–cognitive *ability*. The present study aims to adjudicate between these two explanations for class differences in social cognition. To this end, we assessed class differences in performance on two types of face memory tasks—an *explicit* task in which participants are instructed to commit the faces to memory and an *incidental* task in which participants must spontaneously recall faces to which they were exposed but not instructed to memorize. The explicit task reflects differences in ability as this task represents a context in which all faces are unambiguously relevant to the participant, outweighing the social priors one brings to this situation. The incidental task, in contrast, is sensitive not only to ability differences but also to differences in social priors and the motivational relevance appraisals they trigger. The task assesses if individuals (in the absence of relevance cues from the context) spontaneously appraise other people as relevant and thus habitually commit them to memory. By comparing the two tasks, it is possible to both measure class differences in face memory ability and assess the role of social priors in creating such differences. If class differences in memory performance occur in the incidental task but not in the explicit task, we can infer that varying social priors—rather than levels of memory ability—lead to class-based performance differences.

## The Present Research

We test these hypotheses in three studies. In Study 1 (preregistered), we document a negative association between social class and incidental face memory using a well-validated battery of face memory. In Study 2, using the same face memory battery with a German sample, we replicate the negative association between social class and incidental face memory. In addition, we document that social class is not associated with performance on an explicit face memory task (i.e., when we manipulate faces to be task-relevant by explicitly instructing participants to learn them). In Study 3 (preregistered), we show that the effect generalizes beyond performance on a face memory battery to a context with relevance to a real-world phenomenon: eyewitness identification. We find that higher-class individuals, compared with lower-class individuals, have more selective memory for people seen at a crime scene: The tendency to recall an explicitly relevant person (i.e., a thief) better than an incidental person (i.e., a bystander) in the crime scene is exacerbated for higher-class individuals.

## Study 1

In Study 1, we test our hypothesis that social class is negatively associated with incidental face memory. Before data collection began, we preregistered our design, prediction and analyses plan on the Open Science Framework: https://osf.io/y3bjx. Incidental face memory is measured using a subtask in the Berlin Face Test, an established test battery to measure perceptual and affective facets of face memory (Hildebrandt et al., 2010). First, participants undergo a variety of seemingly unrelated perceptual tasks (e.g., matching a face with another face, judging the gender of a face). The tasks allow participants to be exposed and attend to faces without being instructed to memorize them. Spontaneous memory for faces is then measured by asking participants to take an unexpected test at the end of the test session, in which they are asked to recall the faces that they had previously seen but were not instructed to memorize.

### Participants

A power analysis of a small pilot study revealed that we need 411 participants for 80% power to detect Cohen’s *f*-squared of 0.019. We recruited 399 participants on the Prolific Academic crowdsourcing platform (Peer et al., 2017). In all, 15 participants were excluded from analysis due to missing data on one or more variables of interest or because they participated in the tasks enabling the incidental face memory task multiple times (this could have led to more exposure to the test faces; note that the results remain substantively unchanged if we do not exclude participants). This resulted in a final sample of 384 workers (203 male, 178 female, 3 other), aged 18 to 73 (M = 34.47, *SD* = 12.29). All participants were U.S. nationals. In all, 300 participants identified as White, 30 as African American, 28 as East Asian/South Asian/Asian American, 1 as Native American, and 13 as Latinx, with 12 participants specifying another ethnicity or multiple ethnicities.

### Materials and Procedure

Incidental face memory was assessed using a standardized face test battery task (Hildebrandt et al., 2010) that was programmed in Inquisit software (Inquisit 4.0.0.1, 2012. Seattle, WA: Millisecond Software) and administered online. Face images were standardized gray-scale portraits fit to an ellipse and therefore devoid of any external features such as hairstyle or accessories. Female and male faces were equally represented in all tasks. All faces were of White individuals. All participants read written instructions on the screen and, prior to each task, training trials with feedback were administered. The tasks have been psychometrically tested and validated in several previous studies (Hildebrandt et al., 2011, 2013; Wilhelm et al., 2010).

To enable the incidental face memory task, participants first finished two seemingly unrelated face perception tasks called “delayed nonmatching to sample task” and “gender verification task” (for a detailed description of these tasks, see original article Hildebrandt et al., 2010). During both tasks, participants see the faces they are later asked to recall. Importantly, participants are not instructed to memorize these faces for later recall; rather, they are simply asked to make perceptual decisions about the faces (e.g., whether the face is female or male). After finishing these two tasks, participants complete an unrelated filler task for a duration of approximately 10 min. Incidental face memory is assessed after this filler task. In the incidental face memory task, participants are asked to recall 46 faces they had seen previously. Each previously seen face is shown along with one new distractor face of the same sex. Participants indicate which of the two faces they had seen before by a corresponding key press, resulting in a total of 46 trials. We calculated the probability of correctly identifying a face (i.e., the number of correct identifications out of the 46 faces) as the incidental face memory score.

As preregistered, the participant’s social class was assessed using multiple indicators. For theoretical reasons, our analyses focused on measures of social class that have been used in previous research to assess social class as a form of culture (see Dietze & Knowles, 2016, 2021). First, we used a social-class-category probe prompting participants to place themselves into one of five commonly used class groups (Jackman & Jackman, 1983). The question read: “People talk about social classes such as the poor, the working class, the middle class, the upper-middle class, and the upper class. Which of these classes would you say you belong to?” This social-class item was developed and validated in the United States. To be consistent across all three studies, and to increase the validity of our social-class construct in the German context (Study 2), we supplement this indicator with two other social-class indicators. We assessed participants’ current level of education, and in line with previous research, converted this measure into a binary indicator of college-educated versus not college-educated individuals (Snibbe & Markus, 2005). In addition, given our assumption that social-class cultures are ultimately rooted in different resource ecologies, we administered a questionnaire assessing perceptions of current and childhood resource scarcity (SES scale; Mittal & Griskevicius, 2014) and used these scores as a third indicator of social class, as preregistered. Before answering the social-class questionnaires, participants answered questions to assess standard demographic data (e.g., gender, age, ethnicity, political ideology). We used the same social-class measure across all studies and analyses presented in this article. See https://osf.io/y3bjx for the preregistration and https://osf.io/b3an8/ for full data set, methodology file, and analysis code.

### Results

**Figure 1.**
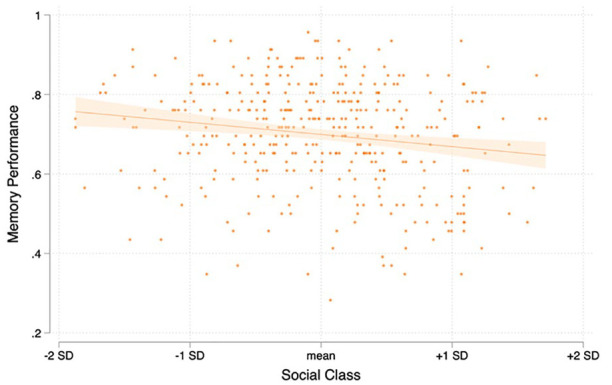
Scatter plot and regression line for the relationships between social class and incidental face memory performance, *r*(382) = −.177, *p* < .001.

We hypothesized that scores on the incidental face memory task would be inversely associated with participants’ social class. To test this, we first examined the bivariate Pearson’s correlation between the social-class composite (α = .699) and incidental face memory scores. Consistent with our preregistered prediction, this negative correlation was significant, *r*(382) = −.177, *p* < .001. See Figure 1 for a visual depiction of the class–incidental face memory relationship in Study 1. As preregistered, we also aimed to test if the relationship is robust against confounds. Given that social class is often confounded with ethnicity—a factor also known to influence face memory performance (Meissner & Brigham, 2001)—we repeated our analysis of the relationship between incidental face memory scores and social class but this time adding dummy-coded ethnicity variables as covariates. Inclusion of these covariates did not substantially change the relationship between social class and incidental face memory scores (B = −0.032, *SE* B = 0.009, *t* = −3.42, *p* = .001, 95% confidence interval [CI]: [−0.050, −0.013]).

### Discussion

In Study 1, we document that social class is negatively associated with incidental face memory such that lower-class individuals exhibit better spontaneous memory for faces than their higher-class counterparts. We hypothesize that the disparities in performance arise from lower-class individuals’ tendency to spontaneously appraise other people as motivationally relevant. Specifically, for lower-class individuals, the mere presence of a face seems to be sufficient to signal importance, bringing about preferential processing, more vivid cognitive representations, and ultimately better recall for faces. While the results of Study 1 are in line with this hypothesis, the results allow for an alternative explanation. A negative association between face memory and social class could be due to differences in perceptual expertise or ability such that lower-class individuals have better face memory in general. Thus, in Study 2, we aim to replicate the documented association between incidental face memory and social class while at the same time examining whether the social class is associated with general face memory ability. In addition, we aim to test whether the results replicate in a different cultural context.

## Study 2

In Study 2, to investigate whether class-based differences in spontaneous memory for faces (Study 1) are due to differences in general *ability* to remember faces, we assess participants’ incidental face memory and explicit face memory within the same testing session. In comparison to incidental face memory, which tests recall for faces without knowing that remembering faces is important for a subsequent task, explicit face memory assesses participants’ ability to recall faces when it is made explicit that it is important to do so. We hypothesize that lower-class individuals will perform better than higher-class individuals on the incidental face memory task (replicating the results of Study 1) but that social class will be unrelated to explicit face memory ability. To test whether the results replicate in a different cultural context, German participants took part in an hour-long laboratory study in which these different facets of face memory were tested.

### Participants

A total of 194 German individuals (104 female, 89 male, 1 other), aged 18 to 52 (M = 28.30, *SD* = 6.46), participated in the study. Participants were either students from the Humboldt-Universität zu Berlin or recruited from the community through a German job platform (i.e., Ebay Kleinanzeigen). Individuals received course credit or cash payment for their participation. Participants’ ethnicity was assessed by asking participants about their place of birth and their parents’ place of birth. 144 participants were born in Germany with both parents also born in Germany (i.e., participants without a migration background). In all, 50 participants indicated that they had migrated to Germany or had at least one parent who migrated to Germany (i.e., participants with a migration background).

### Materials and Procedure

All participants came to the laboratory to take part in the study. To maximize power, we combined data from three existing lab studies. The three studies differed slightly in their overall procedure, but all studies included explicit and incidental face memory tasks as part of a larger face test battery (Hildebrandt et al., 2010). In each of the studies, two tasks assessed explicit memory and one task assessed incidental memory. For the most recent of the three studies (*N* = 61), participants completed a questionnaire assessing demographic information including social-class membership in the lab after they completed the face test battery. We did not have information on social-class membership from participants who took part in the other two studies and thus recontacted these participants to complete a follow-up demographic questionnaire in exchange for 5 Euro online (i.e., from their home). We predetermined that all potential participants would be contacted 3 times to fill out the follow-up questionnaire, twice over email and once over phone. As planned, we ended data collection after these three recruitment cycles. No participants were excluded from the analyses. The final sample consisted of 194 participants (in terms of sample size from each study, the final sample distribution is 49 of 269 participants, 84 of 214 participants, and 61 participants). For the full questionnaire, please see the Supplementary material.

The procedure was very similar to that of Study 1. The face battery tasks were programmed in Inquisit software but the tasks were administered in a laboratory (not online as in Study 1) on a 17-inch-wide PC screen with a refresh rate of 85 Hz. As in Study 1, face images were standardized gray-scale portraits fit to an ellipse and therefore devoid of any external features such as hairstyle or accessories. Female and male were faces equally represented in all tasks and all faces were of White individuals. All participants read written instructions on the screen as well as received verbal instructions by the experimenter according to an experimental protocol. Prior to each task training, trials with feedback were administered and participants could ask questions about the procedure. Participants were instructed to work as quickly and accurately as possible. All tasks have been psychometrically tested and validated in several previous studies (Hildebrandt et al., 2011, 2013; Wilhelm et al., 2010).

The tasks were administered with other tasks of face and object recognition which are beyond the scope of this study. The three relevant tasks were completed in this sequence (with other tasks in between): (a) explicit face memory (called “immediate face memory” in the test battery), (b) incidental face memory, and (c) explicit face memory (called “delayed face memory” in the battery).

The test battery started with the explicit face memory task. Participants were instructed to memorize a matrix of 15 faces in 45 s. Immediately following this learning phase, participants were asked to recall the faces they had learned. On a given trial, two faces appeared—one learned face and one new distractor face of the same sex—and participants indicated which of the two faces they had learned by a corresponding keypress. Each learned face was presented 3 times, each time accompanied by a new distractor face (in one of the three studies, each learned face was presented 5 times). This procedure (i.e., explicit learning followed by immediate recall) was repeated once with a new set of 15 unfamiliar faces, resulting in a total of 90 trials (and 150 trials in one study).

The second explicit face memory task was the last task in the test, about 1 hr after the first explicit face memory task. Here, participants were instructed to recall the faces they were explicitly asked to memorize during the immediate face memory task. Each of the previously learned faces—30 faces in total (i.e., 2 matrices of 15 faces)—appeared once with an unfamiliar distractor of the same sex, resulting in a total of 30 trials. Participants had to indicate the previously learned face by a corresponding button press. We used immediate and delayed face memory performance as separate indicators (eFM_1_ and eFM_2_) in a structural equation model (SEM) representing a latent variable (explicit face memory.) The outcome measure for the SEM was the proportion of correctly recognized faces.

Incidental face memory was assessed in the same way as in Study 1. Again, participants saw 46 faces in unrelated tasks; importantly, they were not instructed to memorize these faces. As in Study 1 and unforeseen by the participants, they were then asked to recall the faces (after finishing an unrelated filler task). On a given trial, one previously seen face was shown along with a new distractor face of the same sex. Participants indicated which of the two faces they had seen before. This resulted in 46 trials which were grouped into three consecutive bins. The proportion of correctly recognized faces in each bin was used as indicators (iFM_1_, iFM_2_, and iFM_3_) for a second latent variable in a structural equation model (incidental face memory).

Participants’ social class was assessed the same way as in Study 1. We used the social-class-category probe prompting participants to place themselves into one of five commonly used class groups (Dietze & Knowles, 2016, 2021; Jackman & Jackman, 1983). To increase the validity of our social-class construct in the German context—the social-class item was developed and validated in the United States—we again supplemented this indicator with participants’ current level of education, and we converted this measure into a binary indicator of college-educated vs. not college-educated individuals (as in Study 1). Finally, we administered the same socioeconomic status scale as in Study 1 assessing perceptions of current and childhood resource scarcity (Mittal & Griskevicius, 2014). We used these three scores as indicators for a latent variable in an SEM called “social class.” Before answering the social-class questionnaires, participants answered questions to assess standard demographic data (e.g., gender, age, place of birth, parent’s place of birth, and political ideology). Participants’ ethnicity was coded as 0 if participants were born in Germany and both parents were also born in Germany (i.e., participants without a migration background) and as 1 if participants indicated that they had migrated to Germany or as having at least one parent who migrated to Germany (i.e., participants with a migration background). See https://osf.io/b3an8/ for full data set, methodology file, and analysis code. This study was not preregistered.

### Results

**Figure 2.**
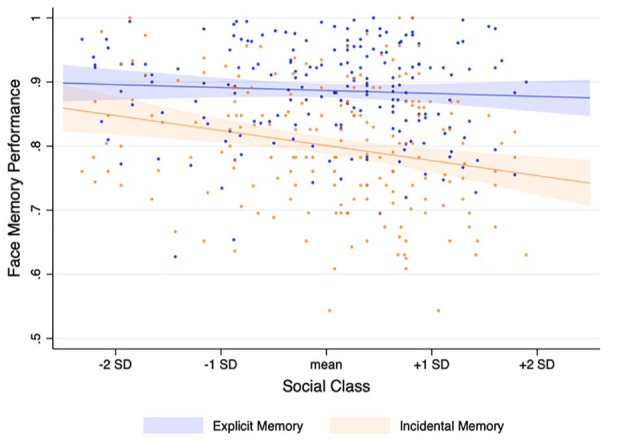
Scatter plots and regression lines for the relationships between social class and explicit memory performance, *r*(192) = −.061, *p* = .397, and social class and incidental memory performance, *r*(192) = −.214, *p* = .003.

As a preliminary test of our hypothesis, we examined bivariate Pearson’s correlations between social class and the two types of face memory; in these analyses, social incidental face memory and explicit face memory were measured using simple composites of their respective indicators. As predicted (and as seen can be seen in Figure 2), no bivariate relationship emerged between social class and explicit face memory, *r*(191) = −.061, *p* = .397, whereas social class significantly and negatively predicted incidental face memory, *r*(192) = −.214, *p* = .003.

Next, we used SEM to estimate latent variables representing explicit and incidental face memory, along with a latent social-class variable. Latent variables have the advantage of accounting for measurement error and method specificity and thus are better suited to investigate associations between psychological constructs than manifest variables. As is customary, we evaluated model fit using the χ^2^ test, the root mean square error of approximation (RMSEA < .08 for acceptable fit), comparative fit index (CFI > .95), and the standardized root mean square residual (SRMR < .08). Latent variables were scaled by fixing their variance to one and their means to zero. All statistical analyses were conducted with the R Software for Statistical Computing using the package *lavaan* (latent variable analyses; Rosseel, 2012).

First, we estimated a two-factor measurement model of explicit and incidental face memory. As described above, there were two indicators for explicit and three indicators for incidental face memory. The correlated factor model fit the data well: χ^2^(4) = 7.580, *p* = .108, CFI = .986, RMSEA = .068, SRMR = .030. All factor loadings were considerable in magnitude and statistically different from zero (explicit face memory: .782 and .809; incidental face memory: .722, .721 and .659). The correlation between incidental and explicit face memory was .476 (*SE* B= .083, *t* = 5.761, *p* < .001) and, hence, shared only 23% of their variance, suggesting that both factors are clearly differentiable constructs. Therefore, we were able to test our hypothesis that incidental but not explicit face memory is negatively correlated with social class.

**Figure 3.**
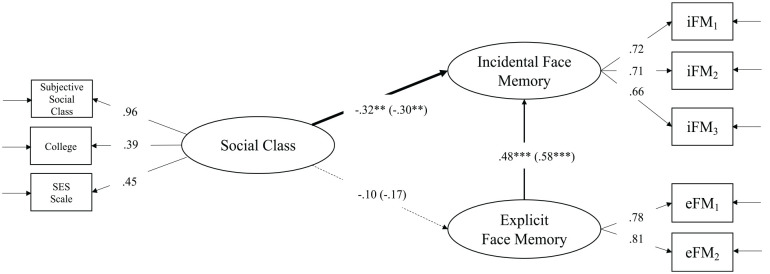
Structural equation model estimating the relationship between social class and explicit vs. incidental face memory. *Note.* iFM_1_, iFM_2_, and iFM_3_ represent the three consecutive blocks of iFM tasks; eFM_1_ and eFM_2_ represent the immediate and delayed eFM tasks. The coefficients in the parentheses are the results when we control for ethnicity and study sample as covariates. iFM = incidental face memory; eFM = explicit face memory. **p < .01. ***p < .001.

Second, we added social class as measured by a latent variable. The two correlated memory factors were predicted by the latent variable social class (see Table S1 in SI for correlations of all variables used in the SEM). The model depicted in Figure 3 had a very good fit to the data: χ^2^(17) = 13.918, *p* = .673, CFI = 1, RMSEA = .000, SRMR = .030. Social class was predictive of incidental face memory (B = −.315, *SE* B = .103, *p* < .01), but not of explicit face memory (B *= −*.095, *SE* B = .087, *p* = .275). To test whether the two regression paths were significantly different from each other, we reestimated the model depicted in Figure 3 with an added constraint—namely, fixing the predictive paths from social class to incidental and explicit face memory to equality. A χ^2^ difference test allowed us to test whether this constraint affected model fit. The equality constraint significantly reduced model fit: χ^2^(1) = 4.21, *p* = .04. Thus, we can conclude that the relationship between social class and incidental face memory is substantially different from the relationship between social class and explicit face memory.

Third, all relationships were estimated by incorporating two further covariates into the model. As in Study 1, the first covariate was ethnicity. The second covariate was “study sample,” as we sampled from three different studies that were not completely overlapping in procedural details (see above). Because the data were merged from three different studies, we used two dummy variables to code the studies. Both latent face memory variables were regressed onto one variable coding ethnicity and two variables coding the study sample. The model fit was satisfactory: χ^2^(35) = 54.56, *p* = .019, CFI = .95, RMSEA = .054, SRMR = .066. Controlling for ethnicity and study sample did not change the relationships between social class and face memory in a meaningful way (see values in parentheses displayed Figure 3).^1^

### Discussion

The results of Study 2 indicate that higher-class individuals exhibit worse spontaneous face memory than lower-class individuals, resulting in a significant negative correlation between social-class and incidental face memory. We also assessed performance on an explicit face memory task—a task that manipulated faces to be relevant by explicitly instructing participants to learn them. As predicted, higher-class and lower-class individuals did not differ in their ability to remember faces when cues in the situation render faces task-relevant. Together, these results suggest that higher-class individuals do not routinely commit a stranger’s face to memory—an effect theorized to be derived from low default relevance appraisals (not lack of ability) among higher-class individuals. Conversely, lower-class individuals appraise other people as motivationally relevant *by default* and thus spontaneously commit faces to memory, regardless of the person’s explicit importance. Finally, we replicated the results of Study 1 in a different cultural context. Participants from Germany and the United States show the same negative association between social-class and incidental face memory, suggesting generalizability of the results across these two national cultures.

## Study 3

Thus far, we have shown that higher-class individuals and lower-class individuals do not differ in their ability to remember faces but that lower-class individuals exhibit better memory for faces that are not explicitly relevant at the time of encoding compared with higher-class individuals. In Study 3, we aim to investigate if this effect can be generalized to a context with relevance to an important real-world outcome—eyewitness identification. In the United States and all over the world, there are countless instances in which people are asked to recall details of a crime scene, not only with regard to who the criminal might have been but also about other people who might have been at the scene or other details in the periphery. Thus, established eyewitness identification paradigms often test memory for different aspects of a crime scene. We use a validated eyewitness testimony task that assesses memory for two types of targets seen in a crime scene video: a thief and a bystander (Murphy & Greene, 2016). The thief in the video is high in relevance (i.e., at the focus of the scene) and the bystander is low in relevance (i.e., on the periphery of the scene). These relevance disparities between the thief and the bystander have been shown to impact face memory—individuals are more likely to correctly identify the thief than the bystander (Murphy & Greene, 2016). Thus, given our finding that high-class individuals exhibit worse memory for faces that are not explicitly relevant at the time of encoding, we hypothesize that higher-class individuals are less likely to correctly identify the bystander from a lineup of possible suspects than lower-class individuals. Given that the thief is considered relevant at the time of encoding, we hypothesize that lower-class and higher-class individuals will not differ in their ability to correctly identify the thief from a lineup. Before data collection began, we preregistered our design, prediction, and analyses plan on the Open Science Framework: https://osf.io/pjxq6.

### Participants

A power analysis of a small pilot study revealed that we need 430 participants for 80% power to detect Cohen’s *f*-squared of 0.025. 451 participants were recruited from the Prolific Academic crowdsourcing platform (Peer et al., 2017). In all, 12 participants were excluded because they failed a simple attention check (i.e., a multiple-choice question that asked participants to simply leave the question blank and to not click any of the answer options) and 1 participant had missing data on the dependent variables. Thus, the final sample consisted of 438 workers (194 male, 232 female, 12 other), aged 18 to 77 (M = 32.01, *SD* = 11.35). All participants were United States nationals. In all, 275 participants identified as White, 35 as African American, 52 as East Asian/South Asian/Asian American, 32 as Latino Hispanic American/Latinx, and 44 participants specifying another ethnicity or multiple ethnicities.

### Materials and Procedure

**Figure 4.**
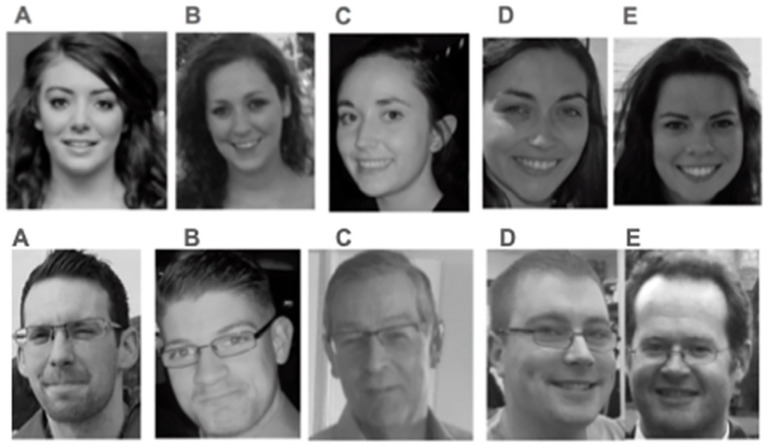
Participants in Study 3 watched a mock crime video and were asked to identify a thief (top row) and a bystander (bottom row) from a lineup of possible suspects. *Note.* Option B is the correct answer for the thief and option E is the correct answer for the bystander.

Participants watch a 51-second-long eyewitness identification video high in cognitive load.^2^ In the video, a thief steals multiple items (e.g., a laptop, money) from an office while a bystander observes the actions of the thief through a window. Both the bystander and the thief are played by White actors, but the thief is a woman and the bystander a man. Before the video starts playing, participants read instructions that prompt them to pay close attention to the video as they will be questioned about the details in the video. After the video was played, participants were asked seven memory questions about objects in the room and other details in the periphery (e.g., “Did you see a stapler on the desk?”; see SI for the full list of questions). Participants were then asked our main dependent variables of interest. First, participants were asked to identify the thief from a lineup of five possible suspects (see Figure 4, top panel). Afterward, participants were asked to identify the bystander from a lineup of five possible suspects (see Figure 4, bottom panel). Responses were coded as 1 if *the participant correctly identified the thief/bystander* and as 0 if *the participant did not choose the correct person* (i.e., if they chose one of the other four suspects).

As preregistered, social class was measured the same way as in Study 1 and Study 2. Before answering the three social-class questionnaires (i.e., social-class-category probe, education, and SES scale), participants answered questions to assess standard demographic data (e.g., gender, age, ethnicity, and political ideology). See https://osf.io/pjxq6 for the preregistration and https://osf.io/b3an8/ for full data set, methodology file, and analysis code.

### Results

The data are nested within participants such that there are 2 trials per participant: 1 response for the thief and 1 response for the bystander. To test our hypotheses, we conducted a mixed-effects logistic regression and examined the effects of target (0 = *bystander*, 1 = *thief*), social class, and the two-way Target × Social-Class interaction on the probability of eyewitness accuracy. We preregistered three covariates: gender, ethnicity, and object memory. Given that the thief is a White woman and the bystander is a White man, we controlled for dummy-coded ethnicity vectors (as in Study 1 and Study 2) as well as dummy-coded gender to adjust for in-group/out-group effects. We controlled for object memory, a continuous variable, to adjust for general memory ability. All three covariates were entered as main effects and were also allowed to interact with the target vector (see Table S2 in the SI for detailed regression table).

Results revealed a marginally significant main effect of target (B = 1.168, *SE* B = 0.679, *z* = −1.72, *p* = 0.86, 95% CI [−0.1641.214, 2.499]), such that White participants’ memory was significantly worse for the bystander than for the target. As expected, we find a nonsignificant main effect of social class (B = −0.20, *SE* B = 0.157, *z* = −1.28, *p* = .200, 95% CI [−0.510, 0.106]), such that social class does not impact *general* face memory ability across the two targets. However, as predicted, we find a significant Target × Social-Class interaction (B = 0.418, *SE* B = 0.212, *z* = 1.970, *p* < .05, 95% CI [0.003, 0.833]).^3^ Examining predicted marginal probabilities, we find that the target effect—the effect that the thief is better remembered than the bystander—is exacerbated for higher-class participants (target effect at +1 *SD* social class: B = 0.312, *SE* B = .052, 95% CI [0.210, 0.415]) and attenuated for lower-class participants (target effect at −1 *SD* social class: B = 0.137, *SE* B= .055, 95% CI [0.028, 0.246]). In sum, we find that higher-class participants’ face memory is more impacted by the explicit relevance of a target (thief vs. bystander) than lower-class participants’ memory and thus, higher-class individuals exhibit more selective memory in a crime scene than their lower-class counterparts.

**Figure 5.**
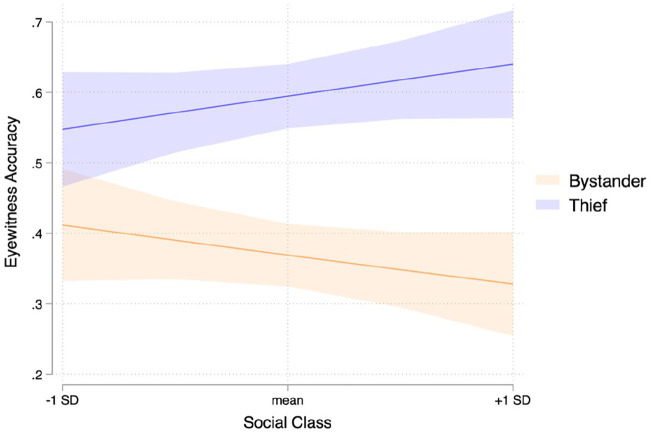
Predicted logistic regression lines and confidence bounds for the relationships between social class and eyewitness accuracy for the thief and bystander.

While these results are in line with our theorizing, the results differ slightly from our preregistration. We predicted a significant negative association between social-class and incidental face memory (i.e., memory for the bystander; orange line in Figure 5) and while this effect is in the predicted direction, it did not reach conventional levels of significance (B = −0.041, *SE* B = 0.032, 95% CI [−0.104, 0.021]).

An alternative explanation for the results of Study 3 (as well as for Study 1 and Study 2) is that lower-class individuals simply commit all incidental information to memory—that is, the class-based memory effect is not unique to incidental faces but generalizes to all incidental objects and details. So in addition to controlling for object memory, we analyzed in an exploratory fashion the bivariate Pearson’s correlation between social-class and object memory. We find a nonsignificant association between the two variables, *r*(436) = −.020, *p* = .674. Thus, the alternative explanation that lower-class individuals remember all types of peripheral information better than their higher-class counterparts can be ruled out. This suggests that the relationship between social class and memory is specific to faces.

### Discussion

In Study 3, we find that higher-class individuals’ eyewitness accuracy was impacted significantly more by a person’s role in a crime scene than lower-class participants.’ For higher-class individuals, seeing a person of high relevance (the thief) compared with an incidental person of low relevance (the bystander) increases the likelihood of correct identification by almost twofold. For lower-class individuals, this form of selective memory is much more attenuated, such that lower-class individuals’ likelihood of correct identification of the thief vs. the bystander only increases by less than half.

Unlike in Study 1 and Study 2, we do not find the hypothesized significant negative association between social-class and memory accuracy for the irrelevant target (i.e., the bystander). This could be due to multiple factors discussed in the general discussion. However, across all three studies, the effect is similar in direction. To synthesize the findings across studies, while also quantifying between-study heterogeneity, we conducted an integrative data analysis (IDA) of the data from Studies 1–3 (Curran & Hussong, 2009).

### Integrative Data Analysis

IDA is similar to meta-analysis but is preferable when all of the relevant data are available to the researcher. In our IDA, we focused on the effect of primary interest: the negative association between social-class and incidental memory for faces. Thus, we retained only trials testing memory for faces without contextual cues to motivational relevance. All trials in Study 1 tested incidental face memory, and so all were retained; in Study 2, we only kept trials probing memory for faces participants were not explicitly instructed to memorize; finally, in Study 3, the item assessing memory for the less-relevant actor in the crime video—the bystander—was retained. To render the dependent variables comparable across studies, we *z*-scored them before combining the data sets.

To estimate between-data set heterogeneity, an IDA can include a study as a random effect (random-effects IDA) or fixed effects (fixed-effect IDA). Because we had only a small number of data sets (3), we chose a fixed-effects approach (Curran & Hussong, 2009). Thus, the study was coded into two vectors representing Study 2 and Study 3 using weighted effect coding (Te Grotenhuis et al., 2017). Unlike traditional effect coding, which would have rendered Study 1 the reference category, weighted effect coding contrasts Study 2 and Study 3 with the sample-size weighted average of all studies. In this approach, the overall effect of social class on incidental face memory in the IDA represents the expected class-memory association for a participant randomly selected from the combined data set.

Using ordinary least squares regression, we regressed incidental face memory (*z*-scored within data set) on the social-class composite, Study 2 vector, Study 3 vector, and the two-way interactions between each study vector and social class. We also included the two demographic variables that were available in all studies—namely, female gender and ethnic/racial outgroup member—which were both weighted effect coded prior to the analysis. These variables were entered as main effects and also allowed to interact with each study vector. Table 1 displays the results of this analysis.

**Table 1.** Results of Integrative Data Analysis of the Association Between Social Class and Incidental Face Memory in Studies 1–3 (Adjusting for Demographic Covariates), *N* = 1,016.

Predictor	*B*	*SE B*	*t*	*p*	95% CI	
					Lower bound	Upper bound
Study 2	−0.025	0.064	−0.399	.690	−0.150	0.100
Study 3	0.013	0.036	0.374	.708	−0.057	0.084
SC	**−0.182**	**0.043**	**−4.223**	**2.631 × 10** ^−5^	**−0.267**	**−0.097**
Study 2 × SC	−0.170	0.087	−1.948	.052	−0.341	0.001
Study 3 × SC	0.093	0.050	1.876	.061	−0.004	0.190
RO	−0.173	0.049	−3.511	4.656 × 10^−4^	−0.270	−0.076
Study 2 × RO	−0.051	0.103	−0.496	.620	−0.252	0.151
Study 3 × RO	−0.082	0.056	−1.481	.139	−0.191	0.027
F	0.108	0.031	3.500	4.861 × −10^−4^	0.047	0.168
Study 2 × F	−0.118	0.063	−1.877	.061	−0.242	0.005
Study 3 × F	−0.011	0.035	−0.320	.749	−0.080	0.058
Intercept	0.011	0.031	0.356	.722	−0.050	0.072

*Note.* Racial outgroup, female, Study 2, and Study 3 were weighted effect coded, with White, male, and Study 1 acting as the reference categories. CI = confidence interval; SC = Social Class; RO = Racial Outgroup; *F* = Female.

The IDA reveals that the negative relationship between social class and incidental face memory is highly robust across studies, B = −.182, *SE* B = .043, *t* = −4.223, *p* = 2.632 × 10^−5^. As the interactions between social class and the Study 2 and Study 3 vectors failed to reach significance, we do not see definitive evidence of heterogeneity across studies—although the negative relationship between social class and incidental face memory was marginally more pronounced in Study 2 and marginally less pronounced in Study 3 relative to the IDA data set as a whole.

Of secondary interest, but in line with past research on the effects of perceivers’ race/ethnicity and gender on face memory (Herlitz et al., 1997; Lewin & Herlitz, 2002), we also find a significant cross-race effect across studies—White participants’ face memory for White faces (White faces were the only stimuli included) was better than ethnic/racial outgroup participants’ face memory for White faces—and a significant positive effect of female gender on face memory across studies.

## General Discussion

Our memory is greatly influenced by what we consider important or relevant to our current goals and well-being. In line with our theorizing that lower-class individuals appraise other people as more relevant, we find that across three studies, lower-class individuals are better at spontaneously remembering faces compared with their higher-class counterparts (IDA). Specifically, we find that lower-class individuals exhibit better incidental memory for faces than higher-class individuals (i.e., better spontaneous recall for faces they saw yet were not instructed to learn; Study 1 and Study 2). This association is not simply due to differences in general memory ability—we find no significant association between social class and explicit memory for faces (Study 2). Higher-class individuals’ tendency to be more selective than lower-class individuals in terms of which faces they remember (i.e., those explicitly flagged as important) also influences performance on an eyewitness accuracy task. We find that, compared with their lower-class counterparts, higher-class individuals exhibit a larger memory discrepancy for people seen at a crime scene. More precisely, the effect that a relevant target (i.e., a thief) is remembered better than an incidental target (i.e., a bystander) is exacerbated for higher-class compared with lower-class observers. In sum, three studies suggest that, as a result of varying degrees of class-based relevance appraisals, an individual’s social class is negatively associated with spontaneous memory for faces. These results are consistent across two cultural contexts (i.e., Germany and the United States).

The current research eliminates a prominent alternative explanation for class-based differences in face memory—and social-cognitive processes more generally: memory ability. Our manipulation of task demands strongly suggests that class differences in face memory are a function of spontaneous appraisals of motivational relevance (or what we have called “social priors”). While our design allows us to isolate the role of motivation (vs. ability), we believe that future research should utilize even more direct measures of relevance appraisals (e.g., event-related potentials measured with an electroencephalogram).

While we found that social class significantly moderates the association between target role and eyewitness accuracy in Study 3, the negative association between a person’s social class and incidental face memory did not reach statistical significance. We see three factors that might have contributed to the result in Study 3. First, the task in Study 1 and 2 was specifically developed to test incidental face memory and thus afforded more experimental control (e.g., minimizing extraneous factors) than the task in Study 3. Second, and related, incidental face memory was assessed with exposure to one face (i.e., one trial per participant) in Study 3 and with 46 trials per participant in Study 1 and 2, substantially increasing the power to detect the effect in Study 1 and 2. Third, the instructions before the video in Study 3 prompted participants to pay close attention—participants knew that what they were seeing would be important for later recall. Thus, while the bystander is less relevant than the thief in the crime scenario, the bystander might still be considered somewhat relevant, at least in comparison to the face images seen during the incidental face memory task in Study 1 and 2. All of these factors might have contributed to the lack of power in Study 3.

Yet, the results of Study 3, especially in conjunction with the results of Study 1 and Study 2, still have important implications for eyewitness testimony. First, while not all forms of eyewitness identification involve incidental face memory, some cases certainly do. As already discussed, one could argue that the exposure to the targets in the video was not solely incidental: A crime unfolded right in front of the observers’ eyes and the instructions made clear that the observers’ memory would be tested. However, eyewitness testimonies in the real world often involve incidental exposure to a person, for example, when an eyewitness encounters a person after or before a crime occurred. This could mean an eyewitness is exposed to a target without knowing that their memory for the person will be important for later recall. Taken together, given that there are different kinds of eyewitness accounts and that our results show that social class is negatively associated with *incidental* face memory, certain eyewitness testimonies might be more influenced by an individual’s social class than others. The evidence seems to suggest that the more incidental the exposure to a target, the more social-class background influences face memory accuracy.

Beyond eyewitness testimony, our results have meaningful implications for everyday life. While much research investigates explicit face memory (i.e., how individuals memorize faces they are explicitly told to learn), people rarely explicitly or intentionally learn faces in the real world. Instead, we are regularly exposed to faces in a spontaneous or implicit manner, not knowing if we will encounter a person again and in what context. Thus, the result that social class is negatively associated with incidental face memory is important because it is ecological valid—it reflects how face memory operates in everyday circumstances. We are exposed to many new faces each day and some we see more than once, for example, people who frequent the same stores or work in the same building. Our memory of a person is influenced by many factors. Face memory research has long documented that group memberships, such as race/ethnicity and gender, is an important determinant of who remembers and who is being remembered. We demonstrate, for the first time, that a perceiver’s social-class membership is another important factor influencing face memory. Across three studies using diverse methodologies, cross-cultural samples, and preregistration, we document a robust lower-class advantage in face memory.

## Supplemental Material

sj-pdf-1-psp-10.1177_01461672221125599 – Supplemental material for A Lower-Class Advantage in Face Memory Click here for additional data file. Supplemental material, sj-pdf-1-psp-10.1177_01461672221125599 for A Lower-Class Advantage in Face Memory by Pia Dietze, Sally Olderbak, Andrea Hildebrandt, Laura Kaltwasser and Eric D. Knowles in Personality and Social Psychology Bulletin

## Notes

1. We initially planned a preregistered replication of Study 2 in the laboratory with another German sample and a modified version of the Berlin Face Test. However, we had to conclude data collection well before reaching our preregistered sample size due to the onset of the pandemic. See supplementary material for details.

2. In the original study, the authors created two videos that were identical in all aspects except in perceptual load—one video was high in perceptual load and the other video was low in perceptual load. In load theory, when the perceptual load is high and cognitive capacity is limited, irrelevant information is less likely to be processed (Lavie, 2005; Lavie & Tsal, 1994). In line with this theory, and validating that the bystander is considered on average to be less relevant than the thief, the researchers found that eyewitness accuracy for the bystander declined significantly when the video was high (vs. low) in perceptual load. The perceptual load had no effect on the accuracy to identify the thief. We only used the video high in perceptual load to avoid ceiling effects.

3. The analysis we preregistered was one utilizing difference scores instead of a mixed logistic regression. If we reanalyze the data in line with the preregistered analysis (see preregistration and supplemental material for details), the result remains substantively unchanged.
