# Supplementary material for: A Lower-Class Advantage in Face Memory
Source: Pers Soc Psychol Bull. 2022 Nov 5;50(2):285–98. doi: 10.1177/01461672221125599 (PMC10860356; doi:10.1177/01461672221125599)
Supplement: sj-pdf-1-psp-10.1177_01461672221125599 – Supplemental material for A Lower-Class Advantage in Face Memory [file sj-pdf-1-psp-10.1177_01461672221125599.pdf]

Supplementary Online Materials

Introduction

In this supplement, we provide additional information on the measures used in each study, results from additional analyses, additional tables, as well as description of a lab study that was terminated due to the pandemic.

Additional Materials and Procedure

Additional Measures Studies 1-3

Social Class. Participants in all three studies filled out the same questionnaires assessing different facets of social class and socioeconomic status. In addition to the social class measures described in the main text—the group-based social class category probe (Jackman & Jackman, 1983), the SES scale (Mittal & Griskevicius, 2014), and the binary education variable (college-educated vs. not college-educated; Snibbe & Markus, 2005)—participants also answered questions concerning their parents' educational attainment, their own income, their household income, and their parents' income while growing up. In addition, as a measure of subjective socioeconomic status, participants rated their perceived standing on a societal "ladder," ranging from 1 (bottom rung) to 10 (top rung ; Adler et al., 2000). For theoretical reasons, we did not include income or the "ladder" as indicators of social class because both measures are context-dependent and/or time-varying, and hence not appropriate measures for predicting cognitive tendencies that are theorized to arise from acculturation into different social classes. The first author of this paper translated all items from English to German in Study 2.

Demographics. Participants in all three studies filled out a short demographic questionnaire including age, gender, and ethnicity. In order to assess ethnicity in Germany, we asked participants about their own and their parents' place of birth.

Additional Measures Study 2

Demographics. In addition to asking participants about their age, gender, and birthplace, we also asked participants about the zip code of the area where they are currently living and the zip code of the area where they spent most of their time while growing up. We also asked them about their political orientation and their religiosity.

Communal Orientation Scale. We asked participants to answer a 14-item communal orientation scale (Clark et al., 1987). The first author of this paper translated the scale from English to German.

Additional Measures Study 3

Object memory. We asked participants about their memory of objects and other peripheral details of the scene with these 7 questions: “What color pants was the thief wearing?”, “There was a cup on the desk. Did it have liquid in it?”, “Was there a desktop computer on the desk?”, “Were any of the windows open?”, “Did you see a stapler on the desk?”, “The thief carried one object out in their hand. Did they carry it in their right or left hand?”, and “Did the person who passed the window come from the right or the left?” We created a continuous variable (called “object memory” in the main text) by summing all correct responses to the 7 questions (0 = *no correct answers*, 7 = *all answers correct*).

Confidence rating. After both target identifications (i.e., thief and bystander), we asked participants how confident they were in their identification with one item: “How confident are

you that the person you selected is actually the thief/bystander in the video?" (1 = *Not at all confident*, 7 = *Extremely confident*).

Additional Results

Additional Analysis Study 1

Binary race covariate. Given the relatively small sample of non-white participants in Study 1, we reanalyzed the data using a dichotomous race variable (0 = non-white participants, 1 = white participants). The results remain substantively unchanged with this coding scheme (B = -0.032, SE B = 0.009, $t = -3.52$, $p < .001$).

Additional Table Study 2

Table S1. Descriptive statistics and bivariate correlations between modeled variables in the SEM (Study 2).

		Mean	SD	1	2	3	4	5	6
1.	Incidental Face Memory	.80	.10	-	-	-	-	-	-
2.	Explicit Face Memory	.89	.07	.39***	-	-	-	-	-
3.	Subjective Social Class	2.74	.83	-.24***	-.08	-	-	-	-
4.	College	0.73	0.45	-.17*	-.01	.38***	-	-	-
5.	SES scale	4.27	1.22	-.13†	-.05	.43***	.18*	-	-

Note. College is coded such that 0 = not college educated and 1 = college-educated. *** p < .001, ** p < .01, * p < .05, † p < .10.

Additional Analysis Study 3

Binary race covariate. Given the relatively small sample of non-white participants in Study 3, we reanalyzed the data using a dichotomous race variable (0 = non-white participants, 1 = white participants). The results remain substantively unchanged with this coding scheme (e.g.,

Target × Social Class interaction: B = -0.300, SE B = 0.152, z = -1.970, p < .05, 95% CI [-0.595, -0.001]).

Preregistered analysis. We preregistered an ordinary least squares (OLS) regression analysis utilizing a difference score instead of the mixed-effects logistic regression reported in the main manuscript (https://osf.io/pjxq6). First, we calculate an eyewitness testimony (EWT) difference score (as preregistered): EWTdiff = EWTbystander - EWTthief. As predicted, we find a negative association between social class and EWTdiff, controlling for participants' gender, participants' race, and participants' memory for objects, B = -.090, SE B = .045, $t = -2.01$, $p <$ 0.05. While both analyses—the preregistered analysis and the analysis reported in the main manuscript—yield the same results, we decided to report the mixed-effects logistic regression because it is considered a more conventional analysis for data that is nested within participants.

Additional Table Study 3

Table S2. Results of the association between social class and eyewitness accuracy in Study 3 (adjusting for demographic covariates).

					95% CI	
Predictor	B	SE B	*t*	*p*	Lower Bound	Upper Bound
Social Class (SC)	0.156	0.112	1.400	0.163	-0.063	0.374
Target	-0.736	0.243	-3.030	0.002	-1.214	-0.259
Target × SC	-0.301	0.153	-1.970	0.049	-0.601	-0.002
East Asian/South Asian/Asian American (AsA)	0.138	0.342	0.400	0.686	-0.533	0.809
African American (AA)	-0.210	0.404	-0.520	0.602	-1.002	0.581
Latinx (L)	0.782	0.457	1.710	0.087	-0.113	1.677

Other Ethnicity/Multiple Ethnicities (O)	-0.121	0.367	-0.330	0.742	-0.841	0.599
Target × AsA	-1.022	0.489	-2.090	0.037	-1.982	-0.063
Target × AA	-0.492	0.563	-0.870	0.382	-1.596	0.612
Target × L	-1.547	0.626	-2.470	0.013	-2.773	-0.320
Target × O	-0.896	0.527	-1.700	0.089	-1.928	0.136
Female (F)	0.355	0.221	1.610	0.108	-0.078	0.788
Target × F	0.099	0.303	0.330	0.743	-0.494	0.692
Object Memory (OM)	-0.140	0.112	-1.250	0.211	-0.360	0.079
Target × OM	0.105	0.153	0.690	0.491	-0.194	0.404
Intercept	0.204	0.175	1.160	0.244	-0.140	0.548

Note. Target was coded such that 0 = thief and 1 = bystander. Ethnicity and gender were dummy-coded with White and male acting as the reference categories. Object memory was coded as a continuous variable with higher scores equaling better memory.

Early termination of a replication study with a modified Berlin Face Test

We included our social class variables in a study that was designed to test the psychometric validity of a modified Berlin Face Test, in which we aimed to test the association between explicit/incidental face memory and social class. This modified Berlin Face Test included a new set of facial identities and faces were presented in color (previous test versions presented black and white stimuli). The new stimuli came from the Berlin Emotion Dataset and the Berlin Face Dataset (Wilhelm et al., 2014).

Unfortunately, the COVID-19 pandemic effectively halted our ability to gather these data in the lab, and we were forced to end data collection prematurely. This resulted in a smaller

sample (N = 154 instead of a preregistered N = 250) than needed to test our hypotheses based on an a priori power analysis. The sample was also skewed in terms of education and age. Participants were younger (ages 18 to 36) and more educated (92% of participants had received a college education) than the general population.

Participants

Participants were 154 adults (55% female, 44% male, 1% other) who were recruited from the community. They were ages 18 to 36 (M = 25.56, SD = 4.13). The participants were mostly German nationals (82%), who identified as White (86%; 8% Black; 7% Asian). The majority said German was their native language (75%).

Materials and Procedure

Incidental face memory and explicit face memory were assessed with a modified version of the standardized face test battery used in Study 1 and Study 2 (described above). The tests were programmed in Psychopy version 3.0.7, with other questions completed via Unipark.de, and testing was conducted in small group settings in a computer lab. Participants' social class was assessed with the same indicators used in Studies 1-3.

Results

We examined bivariate correlations between social class and the two types of face memory. No bivariate relationship emerged between social class and explicit face memory, $r(151) = .11$, $p = .18$ or social class and incidental face memory, $r(151) = -.06$, $p = .50$. Including covariates and using SEM did not substantively change the results; thus, we will not discuss them in detail here. Although we did not find the predicted negative association between social class and incidental face memory, this may be due to both our reduced power to detect a reliable effect in this study and our use of modified versions of the standardized face test battery.

Importantly, when we incorporate the results into the integrative data analysis (IDA; see main manuscript), the inclusion of these additional data does not substantively change our pattern of results: we still find a highly robust negative relationship between social class and incidental face memory across all studies, B = -.139, SE B = .040, $t = -3.44$, $p = .001$.

References

Clark, M. S., Oullette, R., Powell, M. C., & Milberg, S. (1987). Recipient's mood, relationship type, and helping. *Journal of Personality and Social Psychology*, *53*(1), 94–103.

Wilhelm, O., Hildebrandt, A., Manske, K., Schacht, A., & Sommer, W. (2014). Test battery for measuring the perception and recognition of facial expressions of emotion. *Frontiers in Psychology*, *5*, 404.
